# Transmural Flow Upregulates PD‐L1 Expression in Microvascular Networks

**DOI:** 10.1002/advs.202400921

**Published:** 2024-05-02

**Authors:** Zhengpeng Wan, Shun Zhang, Amy X. Zhong, Liling Xu, Mark F. Coughlin, Georgios Pavlou, Sarah E. Shelton, Huu Tuan Nguyen, Satomi Hirose, Seunggyu Kim, Marie A. Floryan, David A. Barbie, F. Stephen Hodi, Roger D. Kamm

**Affiliations:** ^1^ Department of Biological Engineering Massachusetts Institute of Technology Cambridge MA 02139 USA; ^2^ Department of Medical Oncology Dana‐Farber Cancer Institute Boston MA 02215 USA; ^3^ State Key Laboratory of Organ Regeneration and Reconstruction Institute of Zoology Chinese Academy of Sciences Beijing 100101 China; ^4^ Beijing Institute for Stem Cell and Regenerative Medicine Beijing 100101 China; ^5^ Ragon Institute of MGH MIT and Harvard Cambridge MA 02139 USA; ^6^ Parker Institute for Cancer Immunotherapy Boston MA 02215 USA; ^7^ Department of Mechanical Engineering Massachusetts Institute of Technology Cambridge MA 02139 USA

**Keywords:** integrin α_V_β_3_, microfluidic, microvasculature, PD‐L1, tumor microenvironment, transmural flow

## Abstract

Endothelial programmed death‐ligand 1 (PD‐L1) expression is higher in tumors than in normal tissues. Also, tumoral vasculatures tend to be leakier than normal vessels leading to a higher trans‐endothelial or transmural fluid flow. However, it is not clear whether such elevated transmural flow can control endothelial PD‐L1 expression. Here, a new microfluidic device is developed to investigate the relationship between transmural flow and PD‐L1 expression in microvascular networks (MVNs). After treating the MVNs with transmural flow for 24 h, the expression of PD‐L1 in endothelial cells is upregulated. Additionally, CD8 T cell activation by phytohemagglutinin (PHA) is suppressed when cultured in the MVNs pre‐conditioned with transmural flow. Moreover, transmural flow is able to further increase PD‐L1 expression in the vessels formed in the tumor microenvironment. Finally, by utilizing blocking antibodies and knock‐out assays, it is found that transmural flow‐driven PD‐L1 upregulation is controlled by integrin α_V_β_3_. Overall, this study provides a new biophysical explanation for high PD‐L1 expression in tumoral vasculatures.

## Graphical abstract

Elevated endothelial programmed death‐ligand 1 (PD‐L1) in tumor vasculature remains unexplained. Using a microfluidic device, it is found that transmural flow upregulates PD‐L1 in microvascular networks, suppressing CD8 T cell activation. Transmural flow also enhances PD‐L1 expression in tumor microenvironment vessels, controlled by integrin α_V_β_3_. The study provides a biophysical explanation for upregulated PD‐L1 in tumoral vasculature.

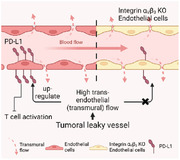

## 1 Introduction

Programmed cell death‐1 (PD‐1)/programmed death‐ligand 1 (PD‐L1) is an immune‐suppressive signaling pathway that restrains the hyperactivation of immune cells, prevents autoimmune diseases, and maintains immune homeostasis.^[^ ^1^ ^]^ However, the PD‐1/PD‐L1 axis is hijacked by cancer cells to escape immune surveillance in tumor microenvironments.^[^ ^2^ ^]^ In addition to cancer cells, endothelial, and other stromal cells in the tumor microenvironment also express higher PD‐L1 than normal tissues, suppressing immune cell anti‐tumor responses.^[^ ^3^, ^4^, ^5^, ^6^, ^7^ ^]^ Tumoral endothelial PD‐L1 is upregulated in response to biochemical factors, such as interferon‐gamma (IFNγ), tumor necrosis factor‐alpha (TNFα), and other cytokines and growth factors secreted by activated immune cells and stromal cells.^[^ ^8^, ^9^, ^10^ ^]^ Endothelial cell (EC) morphology, protein expression, and function are regulated by both biochemical and biophysical cues,^[^ ^11^, ^12^ ^]^ but it is not clear whether any biophysical factors regulate endothelial PD‐L1 expression.

In previous in vitro models of healthy vasculature, luminal flow shear stress on the endothelium has been found to exert a minimal and inconsistent effect on endothelial PD‐L1 expression (Figure S1, Supporting Information). In contrast, tumorr microvasculature is aberrant with highly branched vessels, high tortuosity, reduced flow, and elevated permeability.^[^ ^13^, ^14^ ^]^ Such leaky vessels expose vascular ECs to an abnormally high trans‐endothelial (or “transmural”) flow.^[^ ^15^ ^]^ Transmural flow influences endothelial cell sprouting,^[^ ^16^ ^]^ stimulates lymphatic endothelium to increase fluid transport and DC transmigration,^[^ ^17^ ^]^ and increases both the speed of tumor cell trans‐endothelial migration and migration in the surrounding matrix.^[^ ^18^ ^]^ However, little is known if transmural flow controls vascular endothelial PD‐L1 expression.

Here, we developed a novel microfluidic device to investigate the relationship between transmural flow and endothelial PD‐L1 expression. Transmural flow increased PD‐L1 expression in ECs but not stromal fibroblasts (FBs). Elevated PD‐L1 expression in ECs suppressed CD8 T cell extravasation and IFNγ secretion. Using a model of the tumor microenvironment in which PD‐L1 expression had already been increased by cancer cells, we found that transmural flow further upregulated endothelial PD‐L1 expression. Using blocking antibodies and knock‐out assays, we identified that integrin α_V_β_3_ mediates the transmural flow‐induced endothelial PD‐L1 upregulation. This work introduces a novel biophysical mechanism that contributes to the upregulated PD‐L1 expression in tumor endothelium, which may lead to novel therapies.

## 2 Results

### 2.1 Microfluidic Device for Introducing Transmural Flow in a Microvascular Network (MVN)

**Figure 1:**
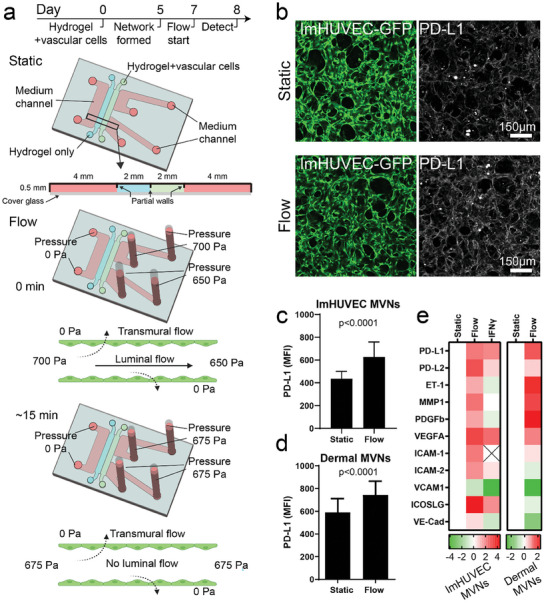
Transmural flow upregulates endothelial PD‐L1 expression. a) Experimental procedure and sketches for investigating PD‐L1 expression in MVNs treated with or without transmural flow. Regarding the flow conditions, initial hydrostatic pressures of 700 Pa in the top right medium channel and 650 Pa in the bottom right medium channel induced a transient luminal flow in the vasculature, which eased after ≈15 min when the culture media in the four syringes reached equilibrium. Transmural flow was driven by the high pressure from the luminal side of vasculature to the basal side (stromal region). b) Representative images of PD‐L1 staining in MVNs treated with (Flow) or without (Static) transmural flow. c,d) Statistical analysis of PD‐L1 mean fluorescent intensity (MFI) in MVNs formed with Immortalized human umbilical vein ECs (ImHUVECs) and lung FBs c) or dermal microvascular ECs and dermal FBs d). Data were collected from at least 30 ROIs across 3 devices. Bars represent mean ± SD. Two‐tailed t‐tests were performed for the statistical comparisons. e) Heatmap of RT‐qPCR results of ImHUVECs and dermal ECs from MVNs treated with or without transmural flow. Fold change was relative to static control. Log2(Fold change) is shown.

A new microfluidic device was designed and fabricated to drive transmural flow through engineered MVNs (**Figure** **1**a; Figure S2, Supporting Information). The main feature of the new design was the two parallel gel channels running the length of the device that enabled the left gel channel to be filled with a cell‐free fibrin gel (Figure S3, Supporting Information) and the right gel channel to be filled with endothelial cells (ECs) and stromal cells in a solution of fibrinogen and thrombin which spontaneously self‐assembled into a MVN. A partial wall was utilized between each channel to provide surface tension, facilitating the retention of the hydrogel precursor before polymerization (Figure 1a). The MVN formed openings to the two medium channels on the right side (Figure S3b, Supporting Information). The top right medium channel serves as MVN inlet, while the bottom right medium channel functions as the MVN outlet (Figure S3b, Supporting Information). Addition of medium to the top right medium channel during medium changes creates a transient hydrostatic pressure difference driving luminal flow to the bottom right medium channel through the MVN (Figure S3b, Supporting Information). Openings in the MVN on the left side, if any, are blocked at the cell‐free fibrin gel. Conversely, adding equal amounts of medium to both right‐side media channels eliminates the driving force for luminal flow leaving the MVN under any desired hydrostatic pressure to drive transmural flow from the MVN and into the left medium channel via the gel‐filled channel.

In the experimental protocol, medium was initially introduced into the right media channels with 50 Pa pressure difference. This induced an initial luminal flow that perfused medium through the entire MVN for a short period (≈15 min, average 6.7 µL min^−1^) before equilibrating and generating transmural flow for 24 h (Figure S3c,d, Supporting Information). This approach ensured full replenishment of the medium within the MVN, delivering nutrients. Varying the mean pressure on the right side relative to that on the left allowed control of the transmural pressure, and hence, the transmural and interstitial flows. The transmural flow was found to be spatially uniform, as demonstrated by fluorescein isothiocyanate (FITC) transport across endothelium under transmural pressure (Figure S3e, Supporting Information). A mean transmural pressure of 675 Pa across the MVN (Figure S3a, Supporting Information) produced an interstitial flow through the left gel channel with a speed of ≈3 µm s^−1^ (Figure S3f–h, Supporting Information).

### 2.2 Transmural Flow Upregulates Endothelial PD‐L1 Expression

Before immunostaining PD‐L1 in MVNs, we validated the specificity of the PD‐L1 antibody (Figure S4, Supporting Information). MVNs with transmural flow expressed more PD‐L1 than MVNs cultured under static conditions (Figure 1b–d). We further confirmed our findings by real‐time qPCR (Figure 1e) and flow cytometry assays (Figure S5a, Supporting Information). In contrast to ECs, transmural flow had no effect on the PD‐L1 expression in stromal FBs (Figure S5b,c, Supporting Information). Last, we found that the endothelial PD‐L1 upregulation required transmural flow treatments for at least 24 h (Figure S5d, Supporting Information).

### 2.3 Increased Endothelial PD‐L1 by Transmural Flow Suppressed T Cell Activation

**Figure 2:**
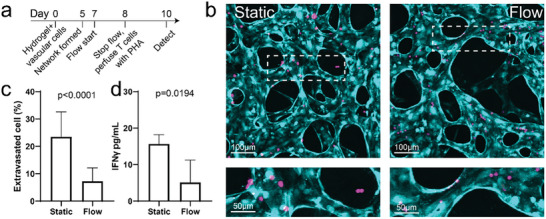
Elevated PD‐L1 expression suppresses T cell activation. a) Experimental timeline for T cell activation assays in MVNs pre‐treated with (Flow) or without (Static) transmural flow. b) Representative images of T cell extravasation in MVNs pre‐treated with or without transmural flow. Dashed rectangle indicates the region of enlarged images below. Cyan, ECs. Magenta, T cells. c) Statistical analysis of extravasated T cell percentage from MVNs treated with or without transmural flow. Data were collected from at least 20 ROIs across 3 devices from each group. d) IFNγ concentration of conditioned media in each group. Data were collected from 3 devices for each group. Bars represent mean ± SD. Two‐tailed t tests were performed for the statistical comparisons.

Following an established protocol,^[^ ^19^ ^]^ we evaluated the effect of transmural flow on cytotoxic T cell (Figure S6, Supporting Information) activation in the MVNs (**Figure** **2**a). Both T cell extravasation and IFNγ secretion were suppressed in MVNs pretreated with transmural flow (Figure 2b–d). This suggests that transmural flow‐driven PD‐L1 expression in ECs may be functional, as it suppressed T cell activation. However, it is possible that there are other mechanisms contributing to T cell suppression in this system, which we have not yet explored.

### 2.4 Transmural Flow Further Upregulates PD‐L1 Expression in Tumor‐Associated Vessels

**Figure 3:**
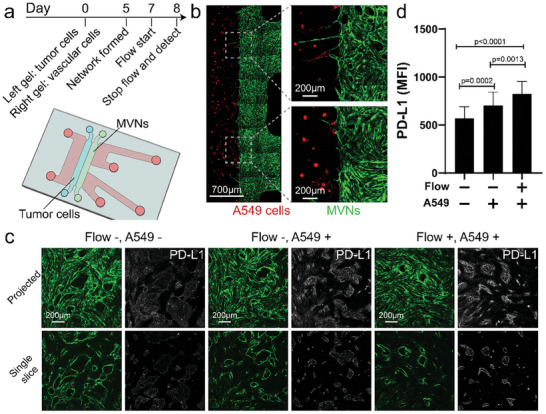
Transmural flow upregulates PD‐L1 expression in tumor‐associated MVNs. a) Experimental procedure for evaluating PD‐L1 expression in tumor‐associated MVNs. b) Representative images of A549 tumor cells (red) and MVNs (green) in the microfluidic devices. c) Representative images of MVNs (green) stained with PD‐L1 antibody (white) when seeded with or without A549 tumor cells in the left gel channel under static condition (Flow ‐) or under transmural pressure (Flow +). d) PD‐L1 MFI of MVNs seeded with or without A549 tumor cells and with (Flow) or without (Static) transmural flow. MVNs were formed with ImHUVECs. Data were collected from at least 30 ROIs across 3 devices. Bars represent mean ± SD. Two‐tailed t tests were performed for the statistical comparisons.

Tumoral blood vessels express more PD‐L1 than normal vasculatures.^[^ ^3^, ^4^, ^5^, ^6^, ^7^ ^]^ Thus, we further tested whether PD‐L1 expression in tumor‐associated vessels could be further increased by transmural flow (**Figure** **3**a,b). A549 lung tumor cells were used to model the tumor microenvironment, as their conditioned medium upregulated PD‐L1 expression in ECs (Figure S2c, Supporting Information).^[^ ^4^, ^20^ ^]^ There was no significant difference in permeability between the MVNs with or without A549 tumor cells cultured in the left gel channel (Figure S7, Supporting Information). Under static conditions, tumoral vessels expressed higher PD‐L1 than control MVNs (Figure 3c). However, the PD‐L1 expression in the tumoral MVNs treated with transmural flow was the highest among all the groups (Figure 3c). This indicates a synergistic effect between transmural flow and tumor cell‐secreted factors.

### 2.5 Integrin α_V_β_3_ Controls Transmural Flow‐Induced Endothelial PD‐L1 Upregulation

**Figure 4:**
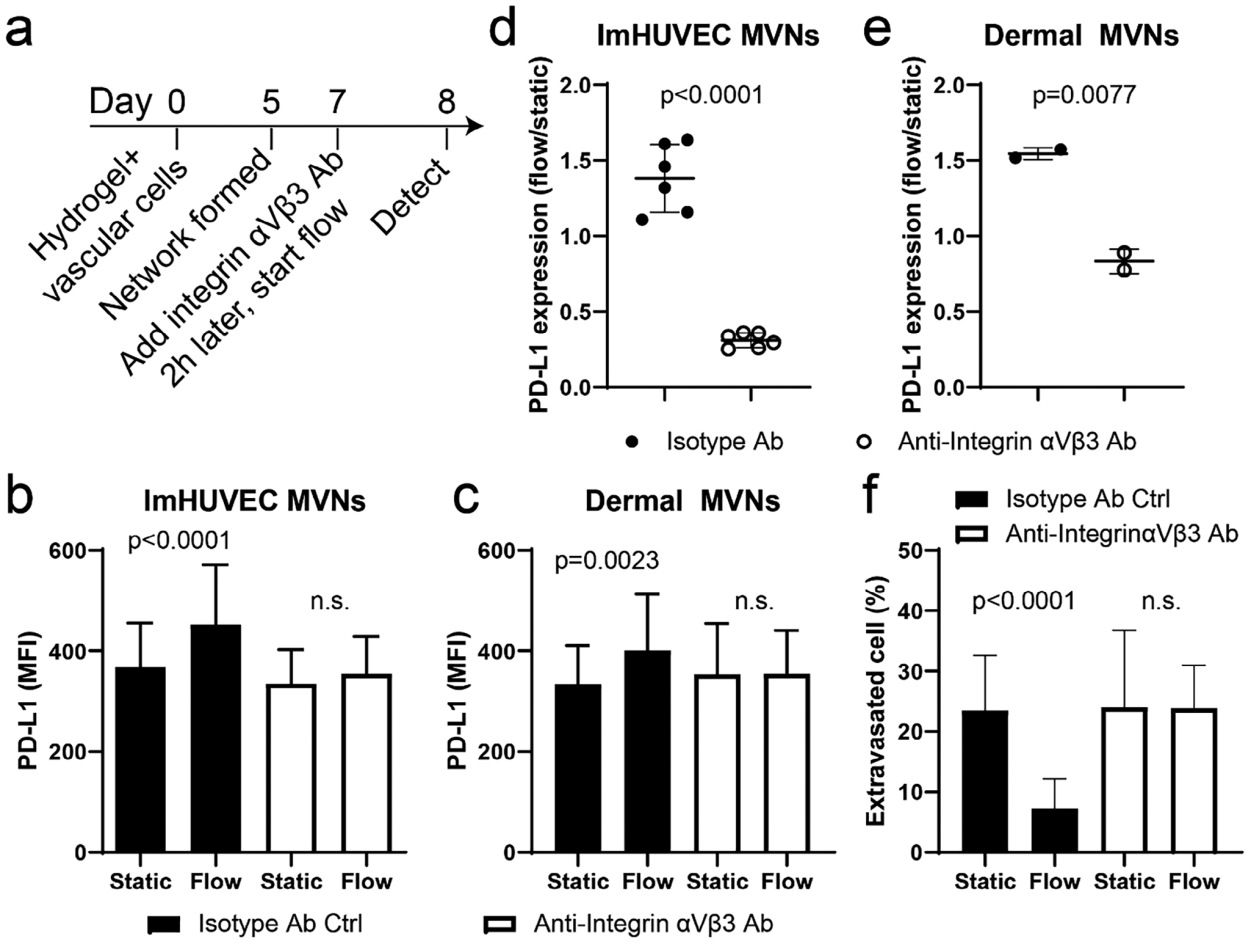
Blocking integrin α_V_β_3_ abolishes transmural flow‐driven PD‐L1 upregulation. a) Experimental timeline for detecting PD‐L1 expression in MVNs which were first blocked with the integrin α_V_β_3_ antibody (Ab) and then treated with transmural flow. b,c) Statistical analysis of PD‐L1 MFI in MVNs made of ImHUVECs b) or dermal microvascular ECs and dermal FBs c). Data were collected from at least 30 ROIs across 3 devices. d,e) Normalized PD‐L1 expression of MVNs made of ImHUVECs d) or dermal vascular cells e). f) Quantification of T cell extravasation at different conditions. Data were collected from at least 20 ROIs across 3 devices. Bars represent mean ± SD. Two‐tailed t tests were performed for the statistical comparisons.

**Figure 5:**
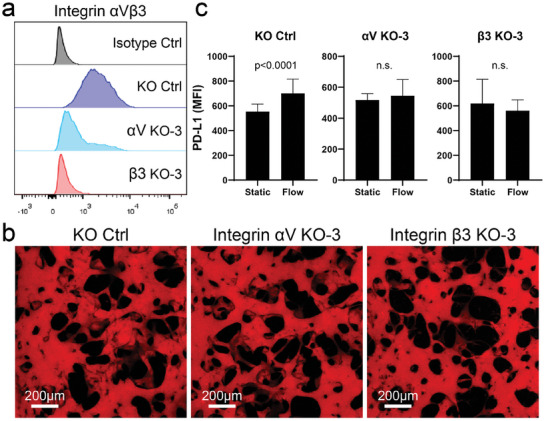
Integrin α_V_β_3_ KO ECs could not upregulate PD‐L1 when subjected to transmural flow. a) Integrin α_V_β_3_ expression in KO control (Ctrl), α_V_ KO‐3, and β_3_ KO‐3 ImHUVECs. b) Representative images of dextran perfused in the MVNs formed with KO Ctrl, α_V_ KO‐3, and β_3_ KO‐3 ImHUVECs indicate that MVNs made with these KO ECs are perfusable. c) PD‐L1 MFI of MVNs made by KO Ctrl, α_V_ KO‐3, and β_3_ KO‐3 ImHUVECs. Data were collected from at least 30 ROIs across 3 devices. Bars represent mean ± SD. Two‐tailed t tests were performed for the statistical comparisons.

Next, we investigated the mechanism of transmural flow‐induced PD‐L1 upregulation in MVNs. Previous studies found that shear stress from intravascular flow activates integrin α_V_β_3_ in ECs and in separate studies integrin α_V_β_3_ has been shown to regulate PD‐L1 expression in cancer cells.^[^ ^21^, ^22^ ^]^ Thus, we tested the possible role of integrin α_V_β_3_ function in transmural flow‐induced PD‐L1 upregulation (**Figure** **4**a). Consistent with this hypothesis, blocking integrin α_V_β_3_ using a specific blocking antibody abolished the transmural flow‐mediated PD‐L1 upregulation in MVNs (Figure 4b–e). Blocking integrin α_V_β_3_ also rescued cytotoxic T cell extravasation from transmural flow‐treated MVNs (Figure 4f). We further confirmed the function of integrin α_V_β_3_ by knocking out (KO) integrin α_V_ or β_3_ in ECs (**Figure** **5**a; Figure S8a–c, Supporting Information). Knocking out either component of integrin α_V_β_3_ reduced the expression of the other component and the integrin α_V_β_3_ dimmer expression on the cell membrane (Figure 5a; Figure S8c, Supporting Information). Both integrin α_V_ and β_3_ KO ECs were able to form perfusable MVNs (Figure 5b) and upregulate PD‐L1 expression in response to IFNγ (Figure S8d, Supporting Information). When treated with transmural flow, KO ECs could not upregulate PD‐L1 (Figure 5c) indicating that integrin α_V_β_3_ is essential for transmural flow‐induced PD‐L1 upregulation in ECs.

## 3 Discussion

In this study, we generated MVNs in microfluidic devices and applied transmural flow to mimic the physical microenvironment created by leaky tumoral vessels in vivo. Endothelial PD‐L1 expression increased with transmural flow over 24 h but not shorter times. In vivo, the hyper‐permeability of tumoral vessels is a chronic condition that may lead to an even greater increase in endothelial PD‐L1 expression than what we have demonstrated in this study.

The microfluidic device used in this study possesses unique features designed for the generation of transmural flow across the endothelium. First, an acellular gel channel blocks the connection between the MVNs and the left medium channel, which functions as a reservoir for collecting transmural flow emanating from the MVNs. Second, the right media channels establish connections with both the upstream and downstream regions of the MVNs, enabling the flow from the top right medium channel to the bottom right medium channel through MVNs. This luminal flow refreshes media in the lumen, ensuring a healthy condition of MVNs. By modulating the mean hydrostatic pressure on the right side of the device relative to that on the left side, this system allows for both controlled transmural and interstitial flows. Notably, this system holds the potential to integrate lymphatic vessels into the left gel channel, providing a platform for engineering a blood‐lymphatic vessel network to study lymph dynamics in future studies.

In this study, we observed suppressed T cell extravasation and IFNγ secretion in the group treated with transmural flow. Our hypothesis posited that elevated PD‐L1 expression in MVNs subjected to transmural flow may be responsible for suppressing T‐cell activation. To test this hypothesis, we utilized PD‐L1 KO ECs. Unexpectedly, our findings revealed that activated cytotoxic T cells killed PD‐L1 KO ECs, resulting in disrupted MVNs (data not shown). Thus, we were unable to establish a direct causal link between transmural flow‐upregulated PD‐L1 and suppressed T‐cell activation. It is possible that other mechanisms within the MVNs pre‐treated with transmural flow may inhibit T‐cell activation. For instance, co‐inhibitory signaling molecules such as PD‐L2,^[^ ^19^ ^]^ CD155,^[^ ^23^ ^]^ and herpes simplex entry mediator (HVEM),^[^ ^24^ ^]^ as well as metabolism‐related factors such as nitric oxide, indoleamine 2, 3‐dioxygenase, and lactate,^[^ ^25^ ^]^ could play a role. Further investigation is required to explore these hypotheses.

For the tumoral vasculature model in this study, only the A549 cell line was tested, based on prior studies demonstrating that conditioned medium from A549 cells effectively upregulates PD‐L1 expression in ECs.^[^ ^4^, ^20^ ^]^ It is worth noting, however, that not all cancer cell types' conditioned media are capable of upregulating PD‐L1 in endothelial cells (Figure S9, Supporting Information). Additionally, other cell types present in the tumor microenvironment, such as cancer‐associated fibroblasts and macrophages, can also increase PD‐L1 expression.^[^ ^26^, ^27^ ^]^ Therefore, developing a more complex tumoral vasculature model to better mimic the tumor microenvironment could be beneficial for studying transmural flow and endothelial PD‐L1 expression in the future.

The level of transmural flow in cancer patients is no doubt highly variable across cancer types and between patients. The transmural flow speed we applied (producing an interstitial flow speed of ≈3 µm s^−1^) is at the higher end of the physiological range.^[^ ^18^ ^]^ It is possible that our flow rate was still lower than the tumoral transmural flow rate in vivo, which has been shown to induce interstitial flow speed in the vicinity of the tumor as high as 10 µm s^−1^.^[^ ^28^, ^29^ ^]^

There is reason to expect that luminal and transmural flow exert different effects on the vascular endothelium. We previously demonstrated that luminal flow, but not transmural flow, promotes tumor cell extravasation potential in vitro.^[^ ^18^ ^]^ Also, the shear stress due to luminal flow acting in healthy, normal tissues is likely to be higher than in the tortuous, somewhat dysfunctional vascular networks in tumors.^[^ ^13^, ^14^ ^]^ Yet, healthy tissues express lower endothelial PD‐L1 than tumor tissues. The induction of PD‐L1 upregulation by transmural flow found in the present study suggests that PD‐L1 expression is specifically regulated by transmural flow. A systematic study comparing the luminal flow and transmural flow is needed to fully elucidate the underlying mechanism(s) and the cause for these apparent contradictions that transmural flow, but not luminal flow, upregulates endothelial PD‐L1 expression.

Integrins and the proteins associated with focal adhesions (e.g., talin, vinculin, and others) are known to act as mechanotransducers, activating intracellular signaling pathways in response to physical stimuli such as shear stress, changes in substrate rigidity, and cell‐cell physical interactions.^[^ ^30^ ^]^ Indeed, integrin activation has previously been demonstrated to result from transmural flow from the basal‐to‐apical side of an endothelial monolayer,^[^ ^31^ ^]^ opposite to the flow direction in the present experiments. In this study, we found that blocking integrin α_V_β_3_ abolishes the apical‐to‐basal transmural flow‐induced PD‐L1 upregulation in MVNs. It remains unclear whether integrin α_V_β_3_ plays the role of a mechanical sensor in this process. Applying transmural pressure to the MVN introduces transmural flow and vessel distention. This distention causes EC stretching that activates integrins, as well as mechanosensing ion channels.^[^ ^32^ ^]^ There may be an overlap between the integrin signaling pathway and the PI3K/Akt/Stat3‐mediated PD‐L1 regulation pathways, potentially leading to the downstream signaling of integrin activation that controls PD‐L1 expression in ECs. This does not preclude the possibility that other mechanosensors respond to transmural flow, and integrin α_V_β_3_ plays the role in the inside‐out signaling pathway that transduces and enhances the signals to activate the PD‐L1 expression pathways similar to the integrin functions in immune cell activation.^[^ ^33^ ^]^

## 4 Conclusion

In summary, using a new microfluidic platform, we found that transmural flow upregulated PD‐L1 expression in ECs through integrin α_V_β_3_. Increased PD‐L1 expression was observed in both normal and tumor‐associated MVNs, and cytotoxic T‐cell activation was suppressed in the transmural flow pre‐conditioned vasculature. This study provides a new biophysical explanation for highly expressed PD‐L1 in tumoral vasculatures, which can now potentially be targeted in novel treatments for cancer immunotherapy.

## 5 Experimental Section

### Cell Culture

Immortalized human umbilical vein endothelial cells (ImHUVECs, P20‐P30)^[^ ^34^ ^]^ and human dermal microvascular endothelial cells (Lifeline Cell Technology) were cultured in VascuLife VEGF Endothelial Medium (Lifeline Cell Technology). Lung FBs (Lonza, P7) and dermal FBs (Lifeline Cell Technology, P7) were cultured in FibroLife S2 Fibroblast Medium (Lifeline Cell Technology). A549 expressing mCherry were cultured in DMEM (Thermo Fisher) with 10% FBS. CD3 pan T cells (Lifeline Cell Technology) were cultured in RPMI1640 with 10% heat‐inactivated FBS along with IL‐2 (18.3 ng mL^−1^) and then stained with anti‐human CD8a antibody (Biolegend) for sorting out cytotoxic T cells using BD FACSAria III.

### Microfluidic Device Fabrication

Microfluidic devices were fabricated and assembled following previous protocols.^[^ ^35^, ^36^ ^]^ In short, a mold for the devices was designed in AutoCAD (Autodesk, Inc.), and imported in Fusion 360 (Autodesk, Inc.) to generate the corresponding tool paths, followed by milling a delrin acetal block with a micro‐computer numerical control (CNC) milling machine (Bantam Tools). PDMS made of a 10:1 ratio of base to cross‐linker (Ellsworth) was cured in the mold at 60 °C for a minimum of 2 h. Devices were sterilized prior to air‐plasma bonding (Harrick) to clean glass coverslips, followed by subsequent curing overnight. This microfluidic device contains two parallel gel channels, a left medium channel, and two right medium channels, each of which can be pressurized to different levels. This arrangement of channels facilitates flow through the MVN in the right‐hand gel region in combination with control over the transmural pressure acting across the vessels in the MVN. Partial walls separate the fluidic channels and serve to confine the liquid gelling solution in the central channel by surface tension before polymerization. The gel channel is 2 mm wide and 0.5 mm tall.

### Vascular Cells Seeding in the Microfluidic Device

ECs and FBs were concentrated in VascuLife containing thrombin (4 U mL^−1^). The cell mixture solution was mixed with fibrinogen (3 mg mL^−1^ final concentration) at a 1:1 ratio and quickly pipetted into the right gel channel of the device through the gel inlet with a final concentration of 6 × 10^6^ mL^−1^ for ECs and 1.0 × 10^6^ mL^−1^ for FBs. Next, VascuLife containing thrombin (4 U mL^−1^) was similarly mixed with fibrinogen (3 mg mL^−1^ final concentration) and loaded into the left gel channel. The device was then placed in a humidified pipette tip box to polymerize at 37 °C for 15 min in a 5% CO_2_ incubator. After the fibrin gel was cured, the VascuLife culture medium was added and changed daily in the device. After 7 days, MVNs were ready for further experiments (Figure S2a, Supporting Information). For RT‐PCR experiments, ECs (6 × 10^6^ mL^−1^) were mixed with fibrinogen and added into the right gel channel, while FBs (1.0 × 10^6^ mL^−1^) were mixed with fibrinogen and loaded into the left gel channel (Figure S2b, Supporting Information). For tumoral MVN formation, ECs and FBs mixture was similarly mixed with fibrinogen and seeded into the right gel channel. A549 tumor cells (1.0 × 10^6^ mL^−1^) were resuspended in VascuLife containing thrombin (4 U mL^−1^) mixed with fibrinogen (total 10 µL) and added into the left gel channel (Figure S2c, Supporting Information).

### MVN Perfusion and Hydrostatic Pressure Setup, Imaging, and Analysis

To confirm the perfusability of the MVN, the culture medium in all the reservoirs was aspirated, followed by addition of 100 µL of 100 µg mL^−1^ 70 kDa MW Texas Red dextran solution (Invitrogen) to the top right channel. If the MVNs were perfusable, the dextran signal was detectable throughout the MVN and the bottom right medium channel (Figure S3b, Supporting Information). Only perfusable MVNs were used for further experiments (success rate over 80%). To apply pressure and introduce transmural flow, syringes were connected to the right medium channels and filled with cell culture medium to indicated heights (70 mm for the top right channel, 65 mm for the bottom right channel). The 50 Pa hydrostatic pressure difference between the top right and bottom right media channels induced a transient luminal flow (reaching equilibrium in ≈15 min), thereby refreshing the medium within the MVNs. The medium in the syringes was refilled every 8–12 h to maintain the transmural pressure for 24 h unless otherwise specified. The static‐conditioned groups were also subjected to the transient luminal flow during medium changes. The average luminal flow was determined by dividing the media volume generating a 50 Pa hydrostatic pressure difference between the top right and bottom right media channels by the time required to reach equilibrium. The average interstitial flow speed produced by the transmural flow was calculated by measuring the accumulated volume of medium in the left gel channel every 8 h. The interstitial flow speed was also measured using fluorescence recovery after photobleaching (FRAP), following the previously published protocol.^[^ ^37^ ^]^ In short, the entire device's matrix was first saturated with FITC‐dextran (150 kDa) by overnight incubation under static culture conditions. Subsequently, hydrostatic pressure was applied by loading culture media containing FITC‐dextran (150 kDa) into the syringes before imaging. For the FRAP imaging setup, a 30 µm diameter spot was bleached in the acellular gel channel, and images were subsequently captured every 0.08 s. Analysis of fluorescence recovery in the bleached spots was conducted using the “frap‐analysis” plugin in MATLAB (MathWorks, version R2022b) to track spot movement velocity, indicative of interstitial flow speed driven by transmural flow.

### MVN Immunofluorescent Staining, Imaging, and Analysis

To detect PD‐L1 expression using immunofluorescent staining, MVNs were fixed by 4% paraformaldehyde. After thorough washing with PBS, MVNs were permeabilized with 2% Triton X‐100 and then blocked with 10% goat serum (Invitrogen). Subsequently, MVNs were stained with PD‐L1 antibody (Biolegend, 1:200 dilution) on a rocker at 4 °C overnight. Next, the microfluidic devices were washed 5 times with PBS on a rocker at room temperature, the MVNs were stained with secondary antibody Alexa Fluor 647‐conjugated goat anti‐mouse IgG (H+L) (Invitrogen, 1:500) on a rocker at 4 °C overnight. After extensive washing, the MVNs were ready for confocal imaging using a confocal laser scanning microscope (Olympus FLUOVIEW FV1200) with a 10× objective. Z‐stack images were acquired with a 5 µm step size. All images shown are collapsed Z‐stacks, displayed using range‐adjusted Imaris software unless otherwise specified. PD‐L1 fluorescent intensity was quantified using ImageJ (NIH, U.S.).

### PD‐L1 Immunofluorescent Staining in 2D EC Monolayer

2D cell staining followed the previous protocols.^[^ ^38^ ^]^ ImHUVECs were cultured in chambered cover glasses (Thermo Fisher Scientific) pretreated by plasma to increase adhesion. Cells were treated with or without 100 U mL^−1^ IFNγ for 24 h, followed by 4% paraformaldehyde fixation for 30 min. After washing with 10 mL PBS, ImHUVECs were incubated with PBS, isotype control antibody (Biolegend, 1:200), or PD‐L1 antibody (Biolegend, 1:200), at 37 °C for 1 h. After washing with 10 mL PBS, these cells were stained with secondary antibody Alexa Fluor 647‐conjugated goat anti‐mouse IgG (H+L) (Invitrogen, 1:500). Images were analyzed by ImageJ (NIH, U.S.).

### RNA Isolation and RT‐qPCR

ECs and FBs were individually seeded in the right and left gel channels, respectively, as illustrated in Figure S2b (Supporting Information). Gels with ECs were cut and used for RNA extraction. Total RNA was isolated using TRIzol reagent (Life Science). Reverse transcription was performed using a high‐capacity RNA‐to‐cDNA Kit (Thermo Fisher Scientific). Quantitative Real‐time RT‐PCR (RT‐PCR) using TB Green Premix Ex Taq II (Tli RNase H Plus) (Takara), was performed with the 7900HT Fast Real‐Time PCR System (Applied Biosystems) and LightCycler 480 II Real‐time PCR Machines (Roche). Glyceraldehyde 3‐phosphate dehydrogenase (GAPDH) was used as the control housekeeping gene. Primers were synthesized by Genewiz. All the primers used in this study are listed in (Table S1, Supporting Information).

### Flow Cytometry

The expressions of PD‐L1, integrin α_V_, β_3_, and α_V_β_3_ were measured by flow cytometry (BD LSR‐II). Cells were stained with APC anti‐human CD274 antibody (Biolegend, 1:100), APC anti‐human CD51 antibody (Biolegend, 1:100), APC anti‐human CD61 antibody (Biolegend, 1:10), or APC anti‐human CD51/61 antibody (Biolegend, 1:100) on ice for 15 min. Cells were then washed twice with MACS buffer and were ready to be examined under flow cytometry. DAPI was used to exclude dead cells.

### Cell Liberation from MVNs

MVNs were treated with or without transmural flow, or treated with IFNγ for 24 h. Next, the right gel channel of the microfluidic device was dissected out. Gels containing ImHUVECs expressing GFP and non‐fluorescent FBs were incubated in 10% Liberase TH Research Grade (Sigma) diluted in DMEM on ice for 30 min. Cells were washed with MACS buffer and stained with PD‐L1 antibody. GFP signals were used to gate the ECs, thus separating them from the FBs.

### T Cell Activation in MVNs

After treated with or without transmural flow, MVNs were perfused with CD8 T cells in the culture medium (Vasculife mixed with RPMI1640 at 1 to 1 ratio) supplemented with 2% phytohemagglutinin (PHA, Thermo Fisher Scientific). Supernatants were collected 48 h after the addition of T cells, for analysis of secreted IFNγ (R&D systems).

### Integrin α_V_β_3_ KO

The guide sequences (Figure S8b, Supporting Information) were integrated into LentiCRISPRv2 blast (Addgene plasmid # 98293). HEK 293T cells were used for producing lentivirus. In short, the plasmids mentioned above were co‐transfected with psPAX2, PMD.G using X‐tremeGENE HP DNA Transfection Reagent (Sigma–Aldrich) for packaging into HEK 293T cells. The supernatant containing lentivirus was collected on days 2 and 3 post‐transfection, followed by Lenti‐X Concentrator (Takara Bio) mediated concentration. ImHUVECs were incubated overnight with the lentivirus in the presence of polybrene (8 µg mL^−1^). Infected cells were recovered for 24 h before selection by blasticidin. KO efficiencies were detected by flow cytometry.

### Treatment of ECs with IFNγ

To test PD‐L1 expression in the integrin α_V_β_3_ KO cells, ImHUVECs were cultured in 6‐well plates and treated with IFNγ (100 U mL^−1^) at cell culture conditions for 24 h before the flow cytometry experiment. PBS was used as vehicle control.

### Statistical Analysis

All error bars are shown as mean ± SD. Statistical analysis was conducted by student's *t*‐test with GraphPad Prism. The confidence level is 95%. Sample numbers and p values are provided in the figures or figure legends.

## Conflict of Interest

R.D.K. is the co‐founder of and holds a significant financial interest in AIM Biotech, a company that produces microfluidic devices. He also receives research support from Amgen, Novartis, Boehringer Ingelheim, AbbVie, Daiichi‐Sankyo, Takeda, Eisai, Visterra, EMD Serono, and Roche. Dr. Barbie is a co‐founder of and holds a significant financial interest in Xsphera Biosciences, and is a consultant for Qiagen/N of One. Dr. Barbie has also received research funding from Novartis, Gilead Sciences, BMS, and Lilly Oncology. Dr. Hodi reports grants and personal fees from Bristol‐Myers Squibb, personal fees from Merck, grants and personal fees from Novartis, Surface, Compass Therapeutics, Apricity, Pionyr, Bicara, Checkpoint Therapeutics, Genentech/Roche, Bioentre, Gossamer, Iovance, Catalym, Immunocore, Amgen, Kairos, Eisai, Rheos, Zumutor, Corner Therapeuitcs, Curis, outside the submitted work; In addition, Dr. Hodi has a patent Methods for Treating MICA‐Related Disorders (#20100111973) with royalties paid, a patent Tumor antigens and uses thereof (#7250291) issued, a patent Angiopoiten‐2 Biomarkers Predictive of Anti‐immune checkpoint response (#20170248603) pending, a patent Compositions and Methods for Identification, Assessment, Prevention, and Treatment of Melanoma using PD‐L1 Isoforms (#20160340407) pending, a patent Therapeutic peptides (#20160046716) pending, a patent Therapeutic Peptides (#20140004112) pending, a patent Therapeutic Peptides (#20170022275) pending, a patent Therapeutic Peptides (#20170008962) pending, a patent THERAPEUTIC PEPTIDES Patent number: 9402905 issued, a patent METHODS OF USING PEMBROLIZUMAB AND TREBANANIB pending, a patent Vaccine compositions and methods for restoring NKG2D pathway function against cancers Patent number: 10279021 issued, a patent Antibodies that bind to MHC class I polypeptide‐related sequence Patent number: 10106611 issued, and a patent Anti‐GALECTIN ANTIBODY BIOMARKERS PREDICTIVE OF ANTI‐IMMUNE CHECKPOINT AND ANTI‐ANGIOGENESIS RESPONSES publication number: 20170343552 pending.

## Supporting information

Supporting Information

## Data Availability

The data that support the findings of this study are available from the corresponding author upon reasonable request.
