# Supplementary material for: Transmural Flow Upregulates PD‐L1 Expression in Microvascular Networks
Source: Adv Sci (Weinh). 2024 May 2;11(26):2400921. doi: 10.1002/advs.202400921 (PMC11234398; doi:10.1002/advs.202400921)
Supplement: Supplementary file 1 — Supporting Information [file ADVS-11-2400921-s001.pdf]

ADVANCED SCIENCE
Open Access

Supporting Information

for *Adv. Sci.*, DOI 10.1002/advs.202400921

Transmural Flow Upregulates PD-L1 Expression in Microvascular Networks

Zhengpeng Wan, Shun Zhang, Amy X. Zhong, Liling Xu, Mark F. Coughlin, Georgios Pavlou, Sarah E. Shelton, Huu Tuan Nguyen, Satomi Hirose, Seunggyu Kim, Marie A. Floryan, David A. Barbie, F. Stephen Hodi and Roger D. Kamm**

Supporting Information

Transmural Flow Upregulates PD-L1 Expression in Microvascular Networks

Zhengpeng Wan, Shun Zhang, Amy X. Zhong, Liling Xu, Mark F. Coughlin, Georgios Pavlou, Sarah E. Shelton, Huu Tuan Nguyen, Satomi Hirose, Seunggyu Kim, Marie A. Floryan, David A. Barbie, F. Stephen Hodi, Roger D. Kamm**

Figure S1

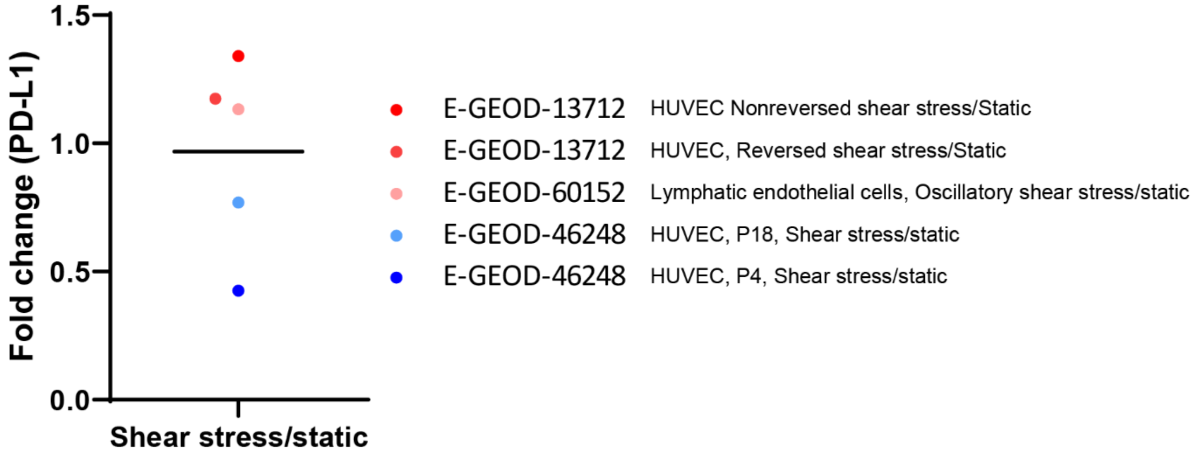

Figure S1. Endothelial PD-L1 expression fold changes between shear stress treatment and static control from published sequencing data. Endothelial Cell Database (EndoDB) was used to search PD-L1 data specific to shear stress treatment. The original data accession numbers are provided (Biostudies https://www.ebi.ac.uk/biostudies/).

Figure S2

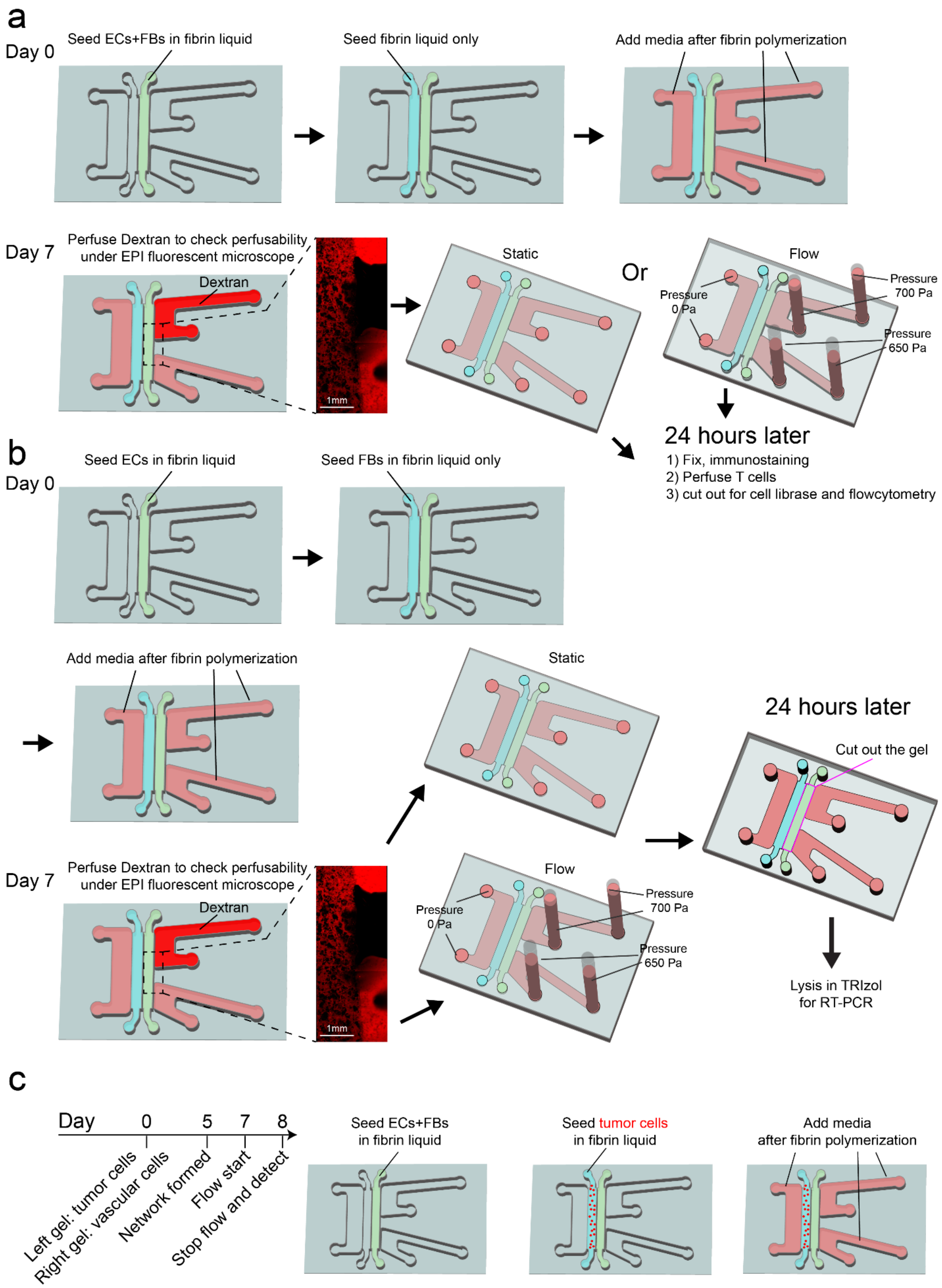

Figure S2. Schematic showing MVN seeding procedures. (a-b) Procedure for MVN formation for immunostaining detection (a) and RT-PCR evaluation (b). (c) Experimental timeline and sketch of tumoral MVN formation.

Figure S3

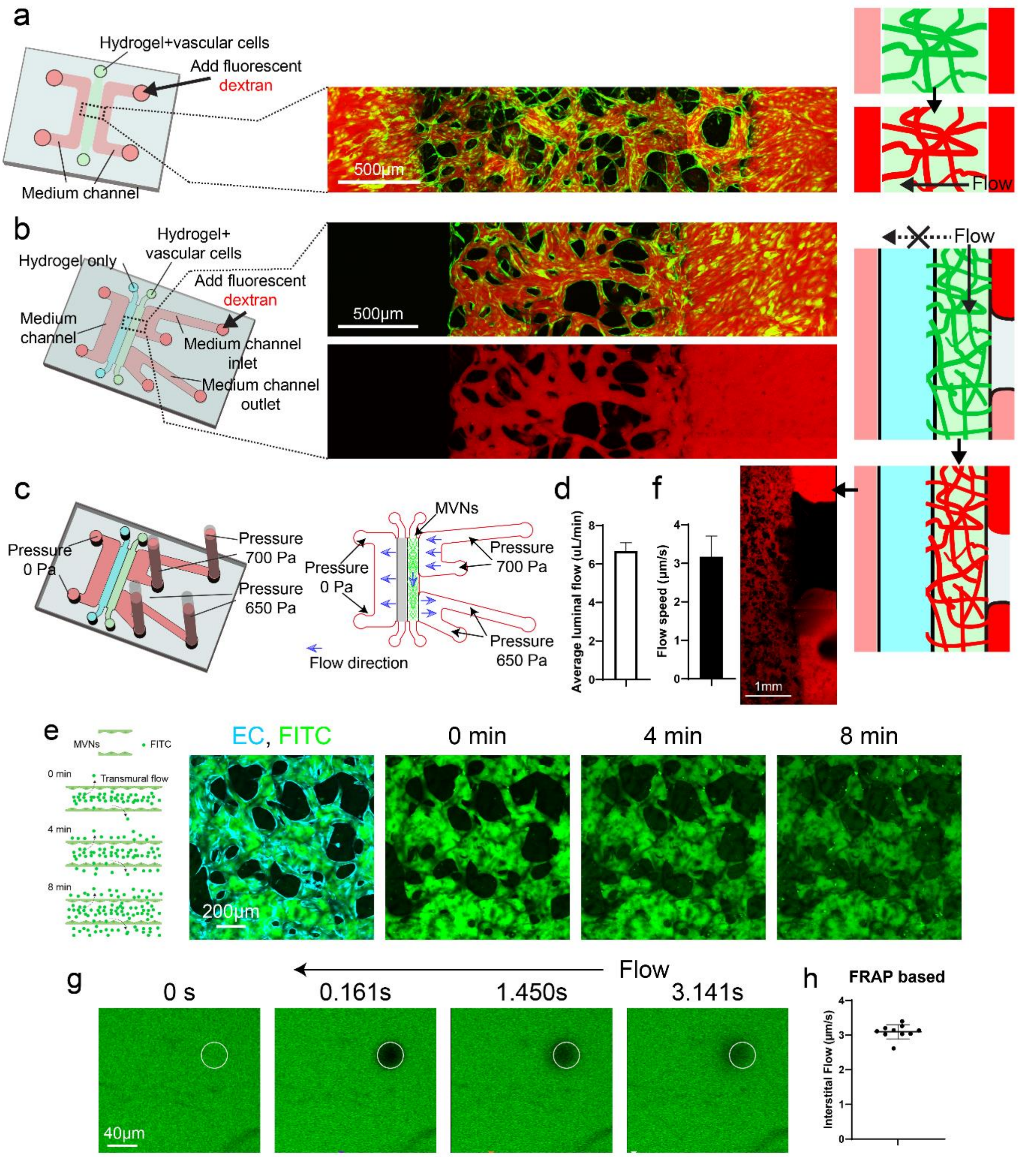

Figure S3. Schematic showing MVN formation and perfusion. (a) Perfusable MVN formed in a single gel device connecting both left and right medium channels. (b) Perfusable MVN was formed in the two-gel channel device, where the left gel blocked the connection between MVNs to the left medium channel, resulting in the flow from the top right medium channel (inlet) to the bottom right medium channel (outlet) through MVN. ECs, Green. Texas Red dextran (70kDa), Red. (c) Sketch showing application of a pressure gradient between the right and left media channels to induce transmural pressure and flow. (d) Average luminal flow. n=6. (e) Confocal imges of FITC (green) trans-endothelial transport under transmural

pressure, demonstrating spatially uniform transmural flow. ImHUVECs expressing BFP were used for MVN formation (cyan). (f) Average flow speed of the interstitial flow towards the left gel region. n=12. (g) FRAP methodology demonstrated through consecutive confocal microscopy images of a round spot (30 μm in diameter) bleached in the acellular gel matrix permeated with FITC-dextran (150 kDa, shown in green) to measure the interstitial flow speed. The scale bar is 40 μm. (h) Flow speed of the interstitial flow towards the left acellular gel region measured by FRAP.

Figure S4

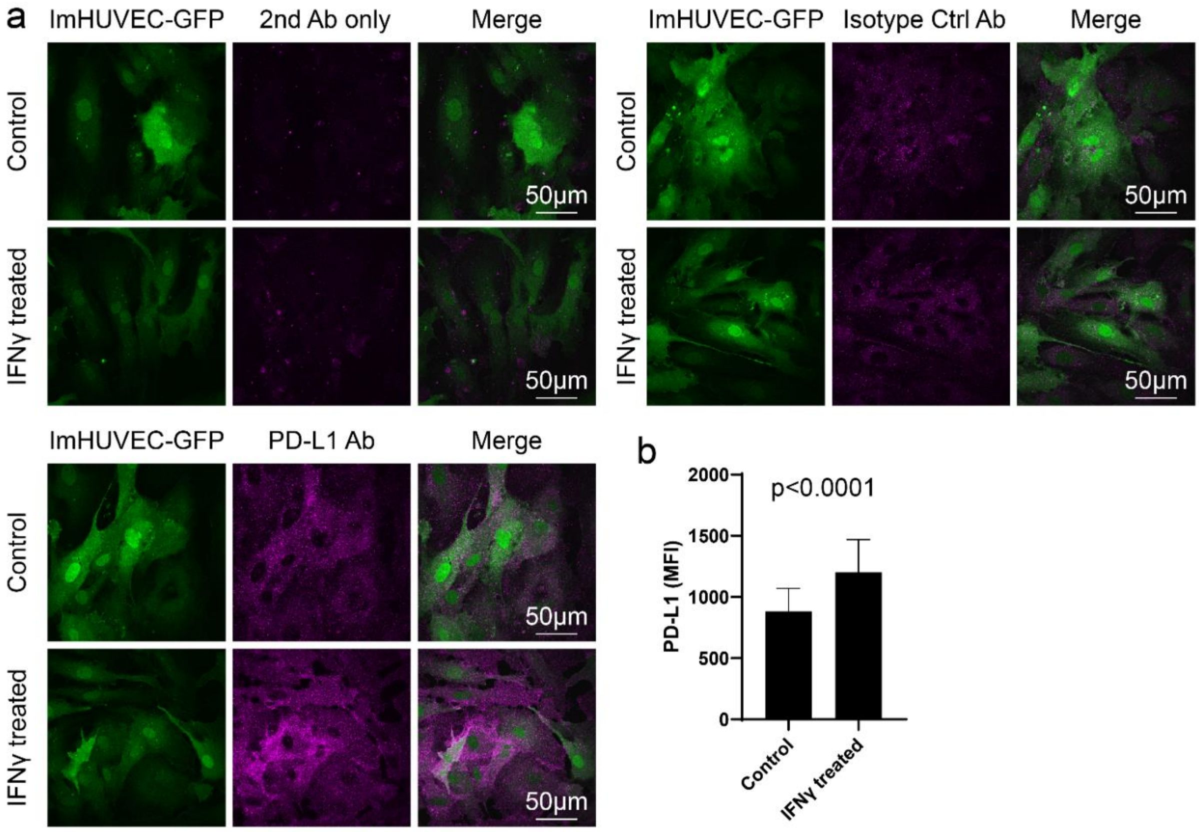

Figure S4. PD-L1 immunofluorescent staining. a) Representative confocal images of PD-L1 immunofluorescent staining in ImHUVECs treated with or without IFNγ. Secondary antibody alone (2nd Ab) and isotype control (Ctrl) Ab were used as controls. b) Statistical analysis of PD-L1 mean fluorescent intensity (MFI) of MVNs treated with or without IFNγ. Data were collected from at least 30 cells. Bars represent mean ± S.D. Two-tailed t tests were performed for the statistical comparisons.

Figure S5

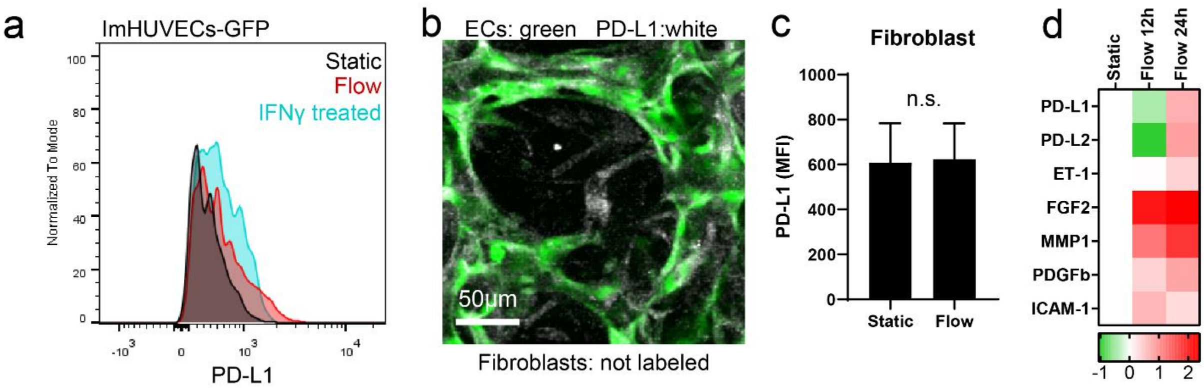

Figure S5. PD-L1 expression of vascular cells in the devices. (a) Flow cytometry data of PD-L1 expression in ImHUVECs isolated from devices treated with IFNγ (100 U/mL) or with (Flow) or without (Static) transmural flow. (b) Representative images of PD-L1 staining in FBs (non-GFP cells) in the devices. (c) PD-L1 MFI of FBs in the devices with (Flow) or without (Static) transmural flow. Data were collected from at least 30 ROIs and 3 devices. Bars represent mean ± S.D. Two-tailed t-tests were performed for the statistical comparisons. (d) RT-PCR data showing gene profile of MVNs under static conditions or with transmural flow for 12h or 24h.

Figure S6

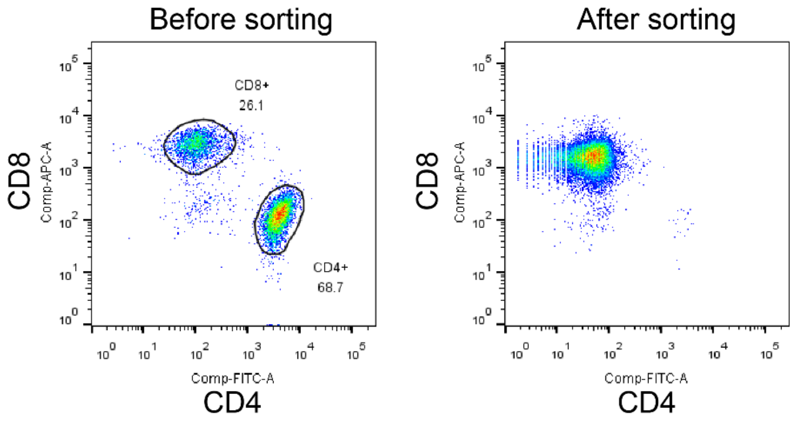

Figure S6. CD8 positive T cells were purified by flow cytometry. After cell sorting, more than 90% of T cells are CD8 positive.

Figure S7

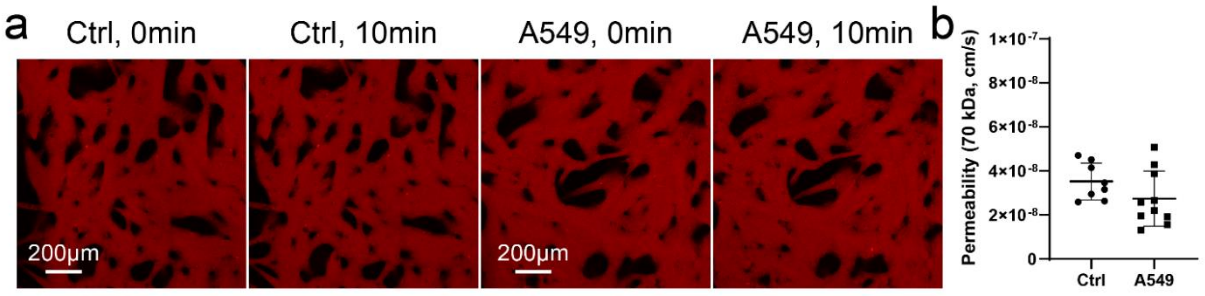

Figure S7. Permeability of Texas-Red dextran (70kDa) in the MVNs cultured with or without A549 cells in the left gel channel. (a) Representative images of Texas-Red dextran (red) in MVNs at 0 min and 10 min. (b) Permeability analysis. Two-tailed t-tests were performed and P=0.1526 (no significant difference).

Figure S8

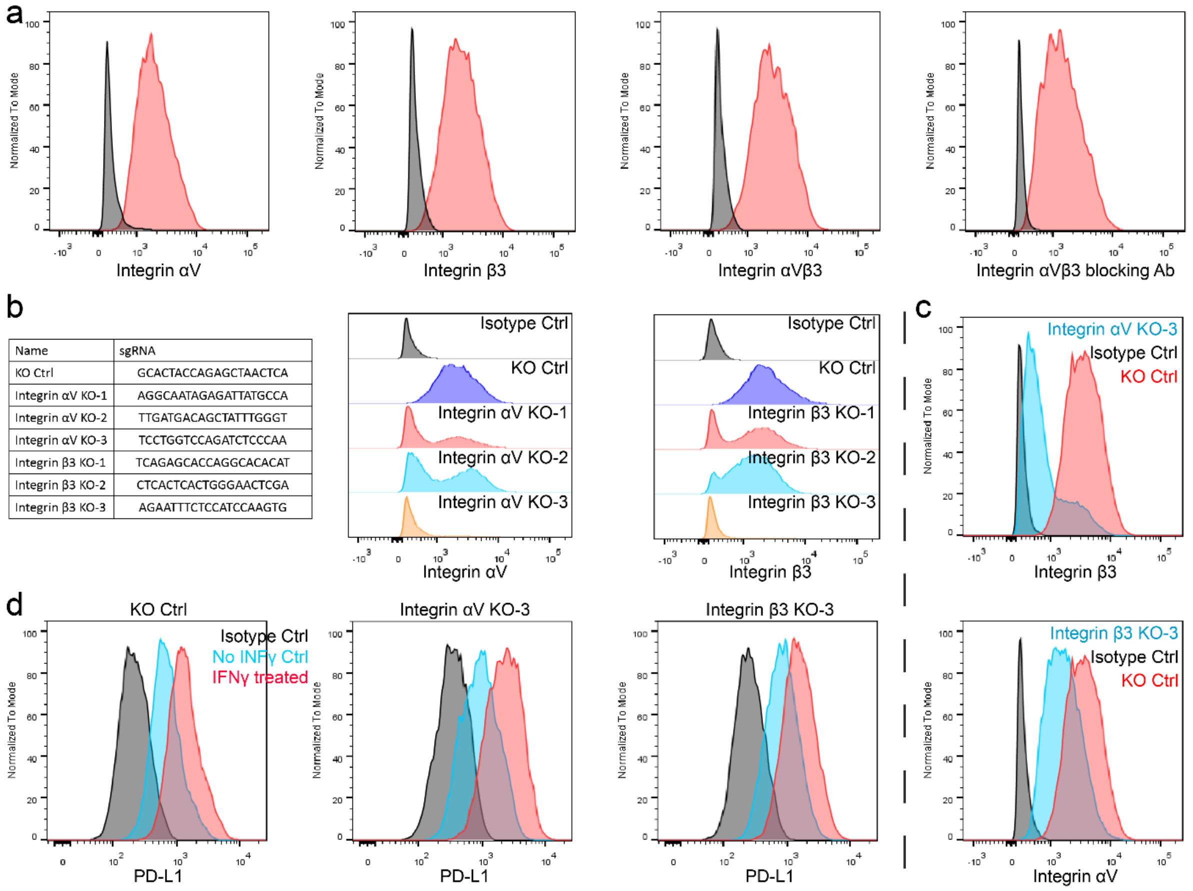

Name	sgRNA
KO Ctrl	GCACTACCAGAGCTAACTCA
Integrin αV KO-1	AGGCAATAGAGATTATGCCA
Integrin αV KO-2	TTGATGACAGCTATTTGGGT
Integrin αV KO-3	TCCTGGTCCAGATCTCCCAA
Integrin β3 KO-1	TCAGAGCACCAGGCACACAT
Integrin β3 KO-2	CTCACTCACTGGGAACTCGA
Integrin β3 KO-3	AGAATTTCTCCATCCAAGTG

Figure S8. Integrin $\alpha_V\beta_3$ KO in ImHUVECs. (a) Expression profile of integrin α_V, β_3, and $\alpha_V\beta_3$ complex in wild type ImHUVECs. Fluorescent Ab targeting integrin α_V, β_3, and $\alpha_V\beta_3$ complex, as well as $\alpha_V\beta_3$ blocking Ab were used. (b) Integrin α_V and β_3 KO guide RNAs and KO efficiencies. (c) Integrin β_3 expression in α_V KO cells and Integrin α_V expression in β_3 KO cells. (d) PD-L1 expression in KO Ctrl, α_V KO, and β_3 KO cells treated with or without IFNγ.

Figure S9

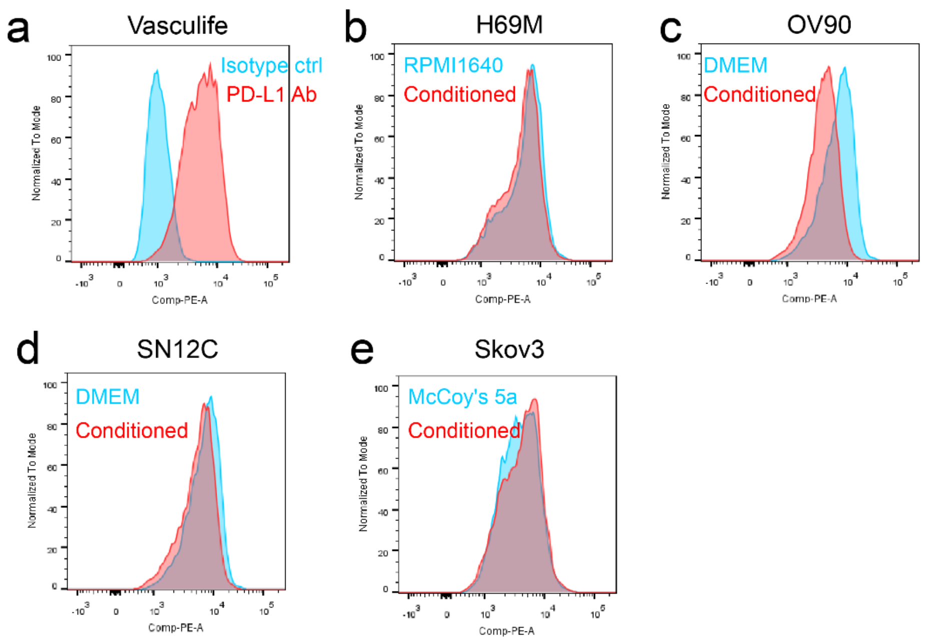

Figure S9. Endothelial PD-L1 regulation by cancer conditioned media. (a) ImHUVECs cultured in Vasculife media, stained with isotype control or PD-L1 antibody. (b-e) PD-L1 expression in ImHUVECs treated with or without conditioned media from H69M (b), OV90 (c), SN12C (d), and Skov3 (e). Respective culture media were used as controls for conditioned media from each cancer cell line.

Table S1. Primers for RT-PCR.

GAPDH-F	ACCACAGTCCATGCCATCAC
GAPDH-R	TCCACCACCCTGTTGCTGTA
PD-L1-F	TGCCGACTACAAGCGAATTACTG
PD-L1-R	CTGCTTGTCCAGATGACTTCGG
PD-L2-F	AGGGAAGTGAACAGTGCTATCTG
PD-L2-R	CTGCAGGCCACCGAATTCTT
Endothelin 1 (ET-1)-F	CTACTTCTGCCACCTGGACATC
Endothelin 1 (ET-1)-R	TCACGGTCTGTTGCCTTTGTGG
MMP1-F	ATGAAGCAGCCCAGATGTGGAG
MMP1-R	TGGTCCACATCTGCTCTTGGCA
PDGFb-F	GAGATGCTGAGTGACCACTCGA
PDGFb-R	GTCATGTTCAGGTCCAACTCGG
VEGFA-F	CGCAGCTACTGCCATCCAAT
VEGFA-R	GTGAGGTTTGATCCGCATAATCT
ICAM-1-F	AGCGGCTGACGTGTGCAGTAAT
ICAM-1-R	TCTGAGACCTCTGGCTTCGTCA
ICAM-2-F	ATGACACGGTCCTCCAATGCCA
ICAM-2-R	GCACTCAATGGTGAAGGACTTGC
VCAM1-F	GATTCTGTGCCCACAGTAAGGC
VCAM1-R	TGGTCACAGAGCCACCTTCTTG
ICOSLG-F	GTTTCACTGCCTGGTGTTGAGC
ICOSLG-R	ACGACGGGCACGCTGAAGTTTG
VE-Cad-F	CTTTTAGGGGATTGTCAGGAGGT
VE-Cad-R	TCCCGATCACTGTACCA ATG
